# Biomonitors of atmospheric nitrogen deposition: potential uses and limitations

**DOI:** 10.1093/conphys/coy011

**Published:** 2018-03-13

**Authors:** Edison A Díaz-Álvarez, Roberto Lindig-Cisneros, Erick de la Barrera

**Affiliations:** 1 Posgrado en Ciencias Biológicas, Universidad Nacional Autónoma de México, Av. Universidad 3000, C.U., Mexico City 04510, Mexico; 2 Instituto de Investigaciones en Ecosistemas y Sustentabilidad, Universidad Nacional Autónoma de México, Ant. Ctra. a Pátzcuaro 8701, Morelia, Michoacán 58190, Mexico

**Keywords:** Atmospheric monitoring, ecosystem saturation, environmental pollution, epiphytic plants, nitrogen content, stable isotopes

## Abstract

Atmospheric nitrogen deposition is the third largest cause of global biodiversity loss, with rates that have more than doubled over the past century. This is especially threatening for tropical regions where the deposition may soon exceed 25 kg of N ha^−1^ year^−1^, well above the threshold for physiological damage of 12–20 kg of N ha^−1^ year^−1^, depending on plant species and nitrogenous compound. It is thus urgent to monitor these regions where the most diverse biotas occur. However, most studies have been conducted in Europe, the USA and recently in China. This review presents the case for the potential use of biological organisms to monitor nitrogen deposition, with emphasis on tropical plants. We first present an overview of atmospheric chemistry and the nitrogen metabolism of potential biomonitors, followed by a framework for monitoring nitrogen deposition based on the simultaneous use of various functional groups. In particular, the tissue nitrogen content responds to the rate of deposition, especially for mosses, whose nitrogen content increases by 1‰ per kilogram of N ha^−1^ year^−1^. The isotopic signature, δ^15^N, is a useful indicator of the nitrogen source, as the slightly negative values (e.g. 5‰) of plants from natural environments can become very negative (−11.2‰) in sites with agricultural and husbandry activities, but very positive (13.3‰) in urban environments with high vehicular activity. Mosses are good biomonitors for wet deposition and atmospheric epiphytes for dry deposition. In turn, the nitrogen saturation of ecosystems can be monitored with trees whose isotopic values increase with saturation. Although given ecophysiological limitations of different organisms, particular studies should be conducted in each area of interest to determine the most suitable biomonitors. Overall, biomonitors can provide an integrative approach for characterizing nitrogen deposition in regions where the deployment of automated instruments or passive monitoring is not feasible or can be complementary.

## Teaser

Atmospheric nitrogen deposition resulting from anthropic activities is a major threat to global biodiversity. Given high costs of deploying automated monitoring networks, this paper makes the case for the use of plant biomonitors, such as mosses, grasses, trees and epiphytes to characterize atmospheric nitrogen pollution.

## Introduction

Nitrogen is one of the essential elements for life and the most abundant in the terrestrial atmosphere, 80% of which is composed of N_2_ (Soderlund, 1976). Due to the high chemical stability derived from its strong triple bond, this molecule can only be divided by processes involving large quantities of energy or through the action of specialized nitrogen-fixing microorganisms (Galloway *et al.*, 2003). For this reason, in the pre-industrial age, more than 99% of the atmospheric nitrogen was unavailable for the great majority of organisms, which lack the enzyme nitrogenase required for fixing N_2_ (White *et al.*, 2012). However, as a result of our growing human population and its associated demand for food and energy, the biologically available nitrogen has more than doubled in the atmosphere over the last century. Agriculture, industry and the use of automobiles are the main sources of a complex of chemical species known as reactive nitrogen (Nr), originated from the splitting of N_2_ (Galloway *et al.*, 2008).

Such an increased deposition of atmospheric nitrogen has adverse effects on biodiversity. Indeed, this form of atmospheric pollution is considered to be the third largest threat to global biodiversity, following only changes in land use and climate (Sala *et al.*, 2000; Payne *et al.*, 2017). In particular, a deposition rate of 10 Kg of N ha^−1^ year^−1^, which has already been recorded for some ecosystems, is sufficient to cause physiological damage in plants (Fenn *et al.*, 2003; Bobbink *et al.*, 2010; Simkin *et al.*, 2016; Payne *et al.*, 2017). Global projections of nitrogen deposition are especially threatening for tropical regions, where it could exceed 25 Kg of N ha^−1^ year^−1^ during the present century (Galloway *et al.*, 2004, 2008; Phoenix *et al.*, 2006).

Implementation of monitoring programs that enable evaluation of the status of this phenomenon and its effects on different ecosystems is thus necessary, especially in the tropics where the most diverse biotas occur. However, the deployment and operational costs of automated air quality monitoring networks may exceed the financial capacity of developing countries. One economical alternative is the use of passive collectors, which are effective in tracking pollution over large areas. Another potential alternative for tracking the nitrogen that enters ecosystems is the use of biomonitor organisms, whose spontaneous occurrence in sites of interest allows an integrative assessment of nitrogen deposition even with a single collection event, as could be during an exploratory field campaign, or in extensive exploration efforts such as national forest surveys. A biomonitor, ‘is an organism that contains information on the quantitative aspects of the quality of the environment’ (Markert *et al.*, 2003). The particular species to be selected in each region of interest (i) should have an ample ecological and geographic distribution, (ii) should be abundant and available throughout the year and (iii) there should be a clear relationship between the variable of interest and the response of the bioindicators (Conti and Cecchetti, 2001).

This paper presents the case for the potential utility of direct measurements of the nitrogen content and isotopic signature of plant tissue for characterizing nitrogen deposition. We start by showing how reactive nitrogen is formed and released to the atmosphere through anthropic activities and discuss the isotopic variation of these chemical species. Next, we explore the advantages and disadvantages of using different types of biomonitors such as mosses and vascular plants, as well as their particular responses to the different forms of nitrogen.

## Reactive species of nitrogen in the atmosphere

**Figure 1:**
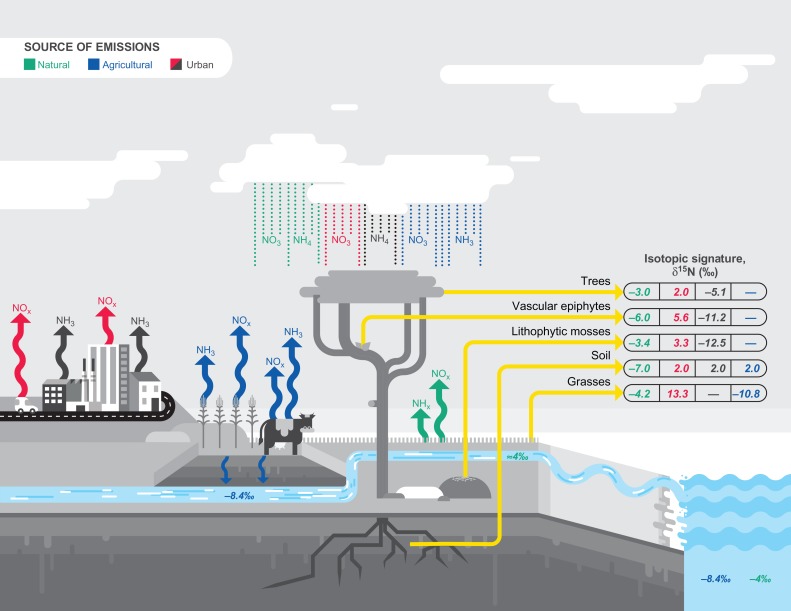
Sources and sinks of nitrogenous emissions. Isotopic composition of different biomonitors including trees, atmospheric plants, lithophytic mosses, grasses and the soil. Green lines and values indicate the isotopic composition of plants from natural environments and ensuing fluxes. Red indicates fluxes derived from exposure to NOx emissions from urban environments, while black NH_3_ from urban environments. Blue tracks the nitrogenous emissions from agriculture and husbandry.

Agriculture releases reactive nitrogen through the volatilization and leaching of nitrogenated fertilizers (Fig. 1; Cameron *et al.*, 2013). In turn, husbandry contributes to such reactive nitrogen through volatilized ammonia gas (NH_3_; Fowler *et al.*, 2013). Industrial activity and motor vehicles also release reactive nitrogen to the atmosphere through the combustion of fossil fuels and other processes that consume large quantities of energy, which break the triple bond of N_2_ and form nitrogen oxides (NOx, i.e. NO and NO_2_; Fig. 1; Galloway *et al.*, 2008).

Such nitrogen oxides and ammonia emitted to the atmosphere are subject to different chemical reactions that lead, for example, to the formation of water-dissolved compounds and gases (NO_3_^−^, NH_4_^+^, HNO_3_), and aerosols [(NH_4_)_2_SO_4_ and NH_4_NO_3_] (Aneja, 2001). These compounds are subsequently transferred to the surface of the earth either as dry deposition, in which the atmospheric gases or aerosols or deposit by gravity, or as wet deposition, in which the nitrogen ions are deposited in fog, snow or precipitation (Fig. 1; Anderson and Downing, 2006; Decina *et al.*, 2017).

## Isotopic composition of atmospheric reactive nitrogen

The isotopic values of reactive nitrogen in the atmosphere have a direct relationship with the source of emission (Box 1). For instance, biogenic emissions of the soil have very negative δ^15^N values between −50‰ and −20‰ (Felix *et al.*, 2013; Felix and Elliott, 2014). Such an ample range of values for gaseous nitrogen species leads to differences in the δ^15^N of the nitrogenous compounds that dissolve in atmospheric water. In particular, the isotopic values of NH_3_ from volatilization of ammonia in the soil and animal wastes, tend to be low, as negative as −40‰ (Freyer, 1978, 1991; Kendall *et al.*, 2007; Felix *et al.*, 2014, 2017). In turn, the δ^15^N for NO_3_^−^ and for NH_4_^+^ range from −15‰ to 15‰, where NO_3_^−^ is usually less negative than NH_4_^+^ (Hoering, 1957; Heaton, 1990; Liu *et al.*, 2012a). In this respect, the negative values observed for the NH_4_^+^ are the result of the very negative NH_3_ reacting in the atmosphere (Felix *et al.*, 2014, 2017). In addition, land use influences the δ^15^N of NH_4_^+^ from wet deposition are less negative in rural areas, ranging from −7‰ to 1‰, than in urban zones where they range from −16‰ to −5‰ (Ammann *et al.*, 1999; Stewart *et al.*, 2002; Xiao and Liu, 2002; Garten, 2006; Liu *et al.*, 2012a; Xiao *et al.*, 2012; Harmens *et al.*, 2014; Sheng *et al.*, 2014).

Box 1: **Stable isotopes and the δ notation**

Isotopes are atoms of an element that have the same number of protons and electrons, but a different number of neutrons; i.e. they are of different atomic mass. Of the known elements, there are at least 300 stable isotopes. Some elements, such as tin, have up to ten, while 21 elements are known to only have one isotope (Sulzman, 2007).

For the case of nitrogen, there are two stable isotopes. ^14^N is the most common and the lightest, with an abundance on Earth of 99.63%. In turn, the heaviest isotope is ^15^N, with a terrestrial abundance of a mere 0.37% (Rosman and Taylor, 1997; Sulzman, 2007). A stable isotope is one that remains energetically stable over time; i.e. it neither emits energy nor decays, as it occurs with radioactive isotopes that gradually mutate towards a more stable state. The better known is the radioactive isotope of carbon,^14^C, which is widely used in archaeological studies (Sulzman, 2007).

Differences in the isotopic composition of some materials are so small that they are reported in parts per thousand (‰), relative to an international standard. The standard used for the isotopic analyses of nitrogen is the N_2_ of the air. The isotopic abundance of a material is determined using the following formula:

$$\delta^{15}N\left(‰\right)=\left(R_{\mathrm{sample}}/R_{\mathrm{standard}}-1\right)\times 1000,$$

where δ^15^N is the isotopic proportion of the sample relative to the standard, *R* is the proportion between the heavy isotope and the light isotope, so that *R*_sample_ is the proportion in the sample and *R*_standard_ is the proportion in the standard (Evans, 2001).

In chemical reactions, the differences in the δ^15^N of the substrate and the product result from a process known as isotopic fractionation through which the lighter isotope is favoured over its heavier counterpart. This process is described by (Δ)

$$\Delta =\delta^{15}N_{s}–\;\delta^{15}N_{p},$$

where δ^15^N_s_ is the isotopic composition of the substrate and δ^15^N_p_ is the isotopic composition of the product (Evans, 2001). One tissue will be more enriched than another when it has a greater proportion of ^15^N, and depleted in the opposite case. For the case of biological reactions, accumulated fractionation is known as isotopic discrimination.

Almost all chemical processes are subject to some degree of isotopic fractionation, in consequence relative abundances of an isotope can reveal the nature of the process from which it comes. Biological organisms are not the exception, all their metabolic reactions reveal their interaction with the environment, allowing track biogeochemical processes. In this case, stable isotopes, particularly of nitrogen, become an excellent integrative tool for understand the organism-environment interactions.

With respect to dry deposition, the isotopic signature of atmospheric NOx is the result of the synergy of various factors. For example, in gasoline and diesel vehicles, both the isotopic composition (positive or negative) and the nitrogen concentration in the fuel interact with the amount of isotopic fractionation during combustion following the mixing of N_2_ with O_2_, which depends on the operation of the engine (Moore, 1977; Felix *et al.*, 2013). The burning of coal and trash can also result in an ample range of δ^15^N values, depending on various factors, including the isotopic composition of the material burned, temperature, pressure and time of the reaction that influence fractionation (Box 1; Moore, 1977; Liu *et al.*, 2012a; Felix *et al.*, 2012, 2013; Felix and Elliott, 2014). For instance, the NOx emitted by electrical energy plants (stationary source) through the combustion of coal has δ^15^N values between 6‰ and 13‰ in South Africa and between 5‰ and 26‰ in China (Heaton, 1990; Li and Wang, 2008). Similarly, the δ^15^N of the combustion of gasoline, diesel, natural gas and the incineration of trash in France yield values of 4.6–7.7‰ (Widory, 2007). In turn, studies of roadside vehicular emissions have δ^15^N of 3.7–15.0‰ (Moore, 1977). In contrast, the combustion of coal and fuel oil in the European country range from −7.5‰ to −5.3‰ (Widory, 2007). And the NOx from the combustion of gasoline in vehicles (mobile source) in South Africa reach isotopic values of between −13‰ and −2‰ (Widory, 2007).

## A framework for biomonitoring atmospheric nitrogen deposition

The use of biomonitors can provide an integrative assessment of ecosystem responses to nitrogenous pollution with consideration of the physiological, ecological and atmospheric conditions of the region of interest (Fig. 1; Sutton *et al.*, 2004; Harmens *et al.*, 2014; Pinho *et al.*, 2017). Species composition and the physiological responses of biomonitor species following experimental manipulations have been amply utilized (Bobbink *et al.*, 2010; Ochoa-Hueso *et al.*, 2011; Lu *et al.*, 2012; Jones *et al.*, 2014). Here, we propose that a better approach to biomonitoring of nitrogen deposition is the determination of total nitrogen content and δ^15^N from plant tissue, which can help characterize both the rate of deposition and the source of the nitrogenous pollution (Sutton *et al.*, 2004; White *et al.*, 2012, Díaz-Álvarez and de la Barrera, 2017). Indeed, various biogeochemical and physiological processes, as well as the determination of nitrogen sources, have been studied through measurements of the isotopic values of soil and plants, including for trees, herbaceous plants, mosses and vascular epiphytes (Emmett *et al.*, 1998; Stewart *et al.*, 2002; Wang and Pataki, 2011; Craine *et al.*, 2015; Díaz-Álvarez *et al.*, 2016; Felix *et al.*, 2016). In this case, the simultaneous consideration of an ensemble of biomonitors of different functional groups is necessary.

### The total nitrogen content indicates the rate of nitrogen deposition

The total nitrogen content of biomonitors can help estimate the rate of atmospheric deposition in an ecosystem. In this case, epiphytic and litophytic mosses are the best potential biomonitors because their tissue nitrogen content is determined by the prevailing atmospheric deposition. Mosses growing on the forest floor are also suitable biomonitors but to a lesser extent, given that the soil can contribute up to 37% of their tissue nitrogen content (Liu *et al.*, 2012a). Estimation of atmospheric deposition is thus possible from the nitrogen content of tissues, which increases by *ca.* 1% (dry weight) for each 10 Kg N ha^−1^ year^−1^ of deposition (Pitcairn *et al.*, 1998; Liu *et al.*, 2008c). This can be observed in natural areas of Europe, where the nitrogen content of mosses ranges between 0.5% and 0.7% and can double in polluted sites (Harmens *et al.*, 2011). However, the nitrogen content of mosses only increases linearly up to a threshold of 20 Kg N ha^−1^ year^−1^, after which it decreases progressively (Pitcairn *et al.*, 2006; Shi *et al.*, 2017). Moreover, when the main form of nitrogen in deposition is NH_4_^+^ such a saturation is reached when this ion exceeds only 12 Kg N ha^−1^ year^−1^ (Pitcairn *et al.*, 1998; Wiedermann *et al.*, 2009). For instance, the nitrogen content of mosses decreases along pollution gradients in China, from 3.0% to 0.9% in urban areas and from 2.3% to 1.6% as pollution increases in rural areas (Liu *et al.*, 2008a,b; Xiao *et al.*, 2010). Given that the inherent nitrogen content of mosses varies amply among species, ranging from 0.1% to 0.5% for different species of pleurocarpus mosses (Pitcairn *et al.*, 1998; Wiedermann *et al.*, 2009; Harmens *et al.*, 2014), it is important to determine dose-response curves for the particular candidate biomonitors in each region of interest.

An important environmental factor that influences the relationship between nitrogen content of the mosses is precipitation. Indeed, the nitrogen content is better correlated with the rate of nitrogen deposition when the annual precipitation is above 1000 mm (Zechmeister *et al.*, 2008). The type of atmospheric deposition (wet or dry) also influences the nitrogen content of mosses. While wet deposition can cause a 0.01% increase in nitrogen content, dry deposition can lead to an increase of nitrogen content between 0.04% and 0.07% for each 1 Kg N ha^−1^ year^−1^, reaching up to 4% in sites with high rates of dry deposition of ammonia, but just up to 1.6% in sites with wet deposition (Hicks *et al.*, 2000; Solga *et al.*, 2005; Pitcairn *et al.*, 2006; Liu *et al.*, 2013a; Harmens *et al.*, 2014).

Vascular plants can also be utilized as biomonitors of the rate of nitrogen deposition, although care must be taken in their consideration as their responses are not linear. For example, the nitrogen content of the epiphytic orchid *Laelia speciosa* (Kunth) Schltr., 1914, amounts to 1.2% (dry mass) under a deposition of 10 Kg N ha^−1^ year^−1^, but 80 Kg N ha^−1^ year^−1^ are required to double the nitrogen content (Díaz-Álvarez *et al.*, 2015). This response has also been observed for seedlings of the tree species *Cryptomeria japonica* (Thunb. Ex L.f) and *Pinus densiflora* (Siebold & Zucc) and for adult individuals of *Pinus resinosa* Aiton. and *Schima superba* (Reinw. ex Blume) (Nakaji *et al.*, 2001; Zhang *et al.*, 2013). In this respect, an increased nitrogen availability often leads to the development of new tissue in vascular plants, rather than to increased levels in the existing cells, thus diluting what otherwise could amount to luxury nitrogen (Taiz and Zeiger, 2002).

Vascular plants can be an excellent complement to mosses for biomonitoring nitrogen deposition. Vascular plants prevail in environments that can be extreme for mosses to prosper, such is the case for urban heat island and arid regions. Additionally, given that vascular plants conform most of the plant cover, they are ideal for using other technologies such as remote sensing which can provide information about biomass and chlorophyll content variations as a result of alterations on atmospheric deposition (Schmidtlein *et al.*, 2012).

### The isotopic composition discerns among natural, agricultural and urban nitrogen sources

The δ^15^N of plants depends on multiple factors, including mycorrhizal associations, form of nitrogen used, soil depth accessed, but most importantly atmospheric sources (Fig. 1; Table 1). Indeed, epiphytic and litophytic plants growing in natural sites without exposure to nitrogenous pollution have δ^15^N that are negative but very close to zero (Wania *et al.*, 2002). In contrast, volatilization and leaching from agricultural and husbandry activities alters the isotopic composition of the vegetation, making it very negative (Craine *et al.*, 2015).

**Table 1:** Isotopic values for different plants from contrasting environments

Life form	Species	Rural δ^15^N	Urban δ^15^N	Reference
Moss	*Braunia* sp.	−3.4‰	3.3‰	Díaz-Álvarez *et al.* (2016)
Mosses	8 species	−12‰	6.0‰	Pearson *et al.* (2000)
Mosses	4 species	−1.4‰	−12.5‰	Liu *et al.* (2008a)
Moss	*Haplocladium microphylum* (Hedw)	−1.3‰	−6.5‰	Liu *et al.* (2008b)
Mosses	4 species	−7.9‰	−3.9‰	Liu *et al.* (2012a)
Annual C_3_ grasses	4 species	−4.2‰	13.3‰	Wang and Pataki (2009)
Herb	*Impatiens* sp.	−1.2‰	−6.1‰	Stewart *et al.* (2002)
Herb	*Calluna vulgaris* (L.)	−8.6‰	0.2‰	Power and Collins (2010)
Vascular epiphytes	8 species	−3.0‰	−10.9‰	Stewart *et al.* (2002)
Epiphytic bromeliad	*Tillandsia recurvata*	−6.0‰	3.0‰	Zambrano *et al.* (2009)
Epiphytic bromeliad	*Tillandsia usneoides*	−11.2‰	−2.2‰	Felix *et al.* (2016)
Epiphytic orchid	*Laelia speciosa*	−3.1‰	5.6‰	Díaz-Álvarez *et al.* (2016)
Tree	*Eriotheca* sp.	−1.6‰	−5.1‰	Stewart *et al.* (2002)
Tree	*Picea abies*	−3.0‰	2.0‰	Ammann *et al.* (1999)

In urban environments the isotopic composition of plants can be positive or negative, depending on the dominant species of reactive nitrogen in the atmosphere (Fig. 1). For instance, in cities where the predominant nitrogen species are gaseous NH_3_ and rain bound NH_4_^+^, the δ^15^N tend to be very negative (Xiao *et al.*, 2010; Liu *et al.*, 2012b; Felix *et al.*, 2013, 2017). This has been documented for urban mosses in China (Liu *et al.*, 2008a,b,c, 2012a,b, 2013b; Xiao *et al.*, 2010) and for urban plants in the vicinity of a fertilizer factory in Brazil, whose δ^15^N reaches −41‰ (Stewart *et al.* 2002; Heaton *et al.*, 2004).

In contrast, the isotopic signature of urban plants from various functional types is positive when NOx is the main source of nitrogen (Fig. 1). This has been documented for different mosses, including *Bryum argenteum* (Hedw) and *Grimmia pulvinata* (Hedw) in London and *Braunia* sp. and *Grimmia* sp. in Mexico City (Pearson *et al.*, 2000; Díaz-Álvarez and de la Barrera, 2017). Such positive values of δ^15^N have also been measured for grasses in the megalopolis of Los Angeles (Wang and Pataki, 2009). The vicinity of roads, where NOx from motor vehicles are emitted, can also determine the isotopic signature in otherwise natural environments, as positive δ^15^N have been measured for the needles of the conifers *Picea abies* (L.) H. Karst. from Norwegian forests (Ammann *et al.*, 1999) and *Pinus edulis* (Engelm) within the Grand Canyon National Park in the USA (Kenkel *et al.*, 2016). A similar response to NOx from motor vehicles has been documented for vascular epiphytes from west-central Mexico such as the orchid *Laelia speciosa* and the bromeliad *Tillandsia recurvata* (L.) (Díaz-Álvarez *et al.*, 2016; Díaz-Álvarez and de la Barrera, 2017).

A group with special potential for biomonitoring nitrogenous pollution in tropical regions is the so called atmospheric plants, a group that includes those epiphytes and lithophytes whose nutrition relies almost exclusive on deposited nutrients. Indeed, given their cosmopolitan distribution atmospheric mosses are widely utilized biomonitors (Markert *et al.*, 2003). Moreover, they are particularly adequate for tropical regions, where they reach their maximum diversity (Cárdenas and Delgadillo, 2009). However, mosses depend on the availability of water for sustaining metabolic activity, thus their monitoring potential is limited to the rainy season. In contrast, succulent epiphytes, especially those with CAM photosynthesis, can be metabolically active throughout the year, thus providing a continuous record of atmospheric deposition regardless of seasonal weather variations (Ammann *et al.*, 1999; Andrade *et al.*, 2007; Zotz *et al.*, 2010). Orchids, for instance, can be found in multiple ecosystems throughout the tropics from sea level up to the subalpine forest above 3500 m (Ernshaw *et al.*, 1987). Atmospheric plants thus, allow a relatively accurate determination of both the source and the magnitude of atmospheric deposition with a very low or null isotopic discrimination given a direct water flux into the cells of mosses (Liu *et al.*, 2012a). In this case, nitrogen is subject to foliar uptake, either by direct influx of gaseous or aqueous nitrogen, i.e. NO, NO_2_, NH_3_ and HNO_3_, directly from the atmosphere during gas exchange or when nitrogen particles are deposited on the plant and dissolved in rain or fog allowing the absorption of the ions NO_3_^−^ and NH_4_^+^ (Hietz *et al.*, 2002; Vallano and Sparks, 2008; Padgett *et al.*, 2009).

Although, atmospheric plants can pick up the isotopic signal of atmospheric deposition, care must be taken when, developing atmospheric biomonitors given the occasional presence of functional roots can obscure the isotopic signal measured from plant tissues (Hietz *et al.*, 2002; Reyes-García and Griffiths, 2009; Liu *et al.*, 2012a). Indeed, epiphytic plants that root in the canopy soil tend to be enriched in ^15^N compared with those that grow on thinner branches, where no substrate accumulation occurs, because the decomposition of the accumulated organic matter produces nitrogenous compounds with δ^15^N close to zero (Wania *et al.*, 2002). Such a canopy soil originated from debris of the phorophyte is depleted in ^15^N relative to the forest soil which tend to accumulate ^15^N as the volatilization and biological uptake of the lighter isotope is favoured (Wania *et al.*, 2002; Liu *et al.*, 2012a; Craine *et al.*, 2015).

### Trees indicate ecosystem nitrogen saturation

The δ^15^N of trees is a good indicator of the state of saturation of atmospheric nitrogen in an ecosystem. The leaves and roots of the trees of N-saturated ecosystems tend to have positive δ^15^N, because saturation increases soil nitrification, a process that involves high rates of isotopic fractionation (Fig. 1; Box 1). In general, plants of ecosystems exposed to low rates of atmospheric deposition tend to present δ^15^N that are negative but close to zero (Craine *et al.*, 2015). However, saturation leads to increased rates of nitrate leaching, which in turn causes saturated soils to become enriched with ^15^N, thus their δ^15^N can become positive. Saturation also makes the relation between foliar δ^15^N and nitrification closer than that between foliar δ^15^N and the δ^15^N of the nitrogen deposition (Ollinger *et al.*, 2002; Pardo *et al.*, 2006; Emmett, 2007). The opposite occurs for translocated nitrogen as a series of isotopic fractionations occurs as it moves from the roots to the branches to the leaves, because a series of enzymes such as nitrate reductase, nitrite reductase and glutamine synthetase are involved in nitrogen transformation, and each one has its own amount of discrimination (Evans, 2001).

Associations with mycorrhizal fungi also influence the δ^15^N of the plants, and trees in particular, having the potential to alter both the nitrogen relations of the plants and the isotopic signature of the assimilated nitrogen (Craine *et al.*, 2009, 2015). Under natural conditions (lower rates of atmospheric deposition), mycorrhizae supply their hosts with nitrogen that is depleted in ^15^N (Emmett *et al.*, 1998). However, saturation can induce species turnover within the mycorrhizal community, from species with high amounts of isotopic discrimination against ^15^N to species with low discrimination, contributing to the isotopic enrichment of the plants and the homogenization of the isotopic signature of the ecosystem (Emmett *et al.*, 1998; Craine *et al.*, 2009; Sheng *et al.*, 2014).

## Metabolic limitations of biomonitors

Biomonitors can become useful tools for detecting nitrogenous pollution over wide areas of terrestrial ecosystems. However, organismal responses are constrained by enzymatic processes. For brevity, this discussion is restricted to the metabolic limitations of mosses, which assimilate NH_4_^+^ to a greater extent when supplied simultaneously with NO_3_^−^. Likewise, these organisms preferentially assimilate organic compounds such as amino acids. For example, under simultaneous application of glycine with NH_4_^+^ and NO_3_^−^, assimilation of this amino acid is up to two times greater than that of the nitrate (Wanek and Pörtl, 2008; Wiedermann *et al.*, 2009). The main reason for this is the high energetic cost of assimilation of NO_3_^−^, which requires two consecutive reactions. In the first, NO_3_^−^ is reduced to NO_2_^−^ by the enzyme nitrate reductase, consuming two electrons in the process. In the second, NO_2_^−^ is reduced to NH_4_^+^ by nitrite reductase, using six electrons (Heldt and Piechulla, 2011).

Nitrate reductase can be inhibited by assimilation of NH_4_^+^ from atmospheric deposition when the ratio between NH_4_^+^ and NO_3_^−^ is high (Liu *et al.*, 2012a). Furthermore, high rates of atmospheric deposition can reduce or even completely inhibit nitrate reductase activity, whether it is due to the strong relationship between NH_4_^+^ and NO_3_^−^, or to the increased concentration of NO_3_^−^ in the deposition of nitrogen. Indeed, while certain concentrations of NO_3_^−^ are necessary to stimulate nitrate reductase synthesis and activity, an excessive amount of the ion exerts a negative feedback on the enzyme (Heldt and Piechulla, 2011). For this reason, when atmospheric deposition reaches 10 Kg N ha^−1^ year^−1^, a significant reduction is observed in the assimilation of NO_3_^−^ and, on exceeding 30 Kg N ha^−1^ year^−1^, the nitrate reductase in the mosses is totally suppressed (Gordon *et al.*, 2002; Forsum *et al.*, 2006; Liu *et al.*, 2012a,b). High concentrations of atmospheric NOx (greater than 63 nL L^−1^) cause suppression of nitrate reductase in mosses of different anthropic environments. Exposure to NO causes nitrate reductase activity to decrease within 24 h, while exposure to NO_2_ causes such an activity reduction over 21 days leading to the complete loss of inducibility of nitrate reductase even when NO_3_^−^ is available (Morgan *et al.*, 1992; Forsum *et al.*, 2006; Liu *et al.*, 2012a,b).

Reduced assimilation of nitrate forces the mosses to assimilate other nitrogenated compounds in the atmospheric deposition, the different isotopic values of which are presented in Table 1. As a consequence, inhibition of nitrate reductase can cause variation in the isotopic values of moss tissues and can make determination of the source of the nitrogen observed in the tissue differ from the true source by up to 21% (Liu *et al.*, 2012a,b).

Thus, inhibition of nitrate reductase can cause a discrepancy between the nitrogen content of the mosses and the rate of atmospheric deposition on the site they inhabit. This can occur because nitrate that is deposited on the mosses can be partially assimilated or may not be assimilated at all. This will depend on the degree of inhibition of nitrate reductase. Consequently, part of the deposition (which contains the nitrate) will not be accurately recorded. In this case, estimation of atmospheric deposition could be more accurate in mosses when the ratio between NH_4_^+^ and NO_3_^−^ is higher than in deposition with low NH_4_^+^ and NO_3_^−^ ratios (Liu *et al.*, 2012a). It has been observed that the nitrogen content of mosses is lower under wet than under dry deposition (Pitcairn *et al.*, 2006; Liu *et al.* 2012a,b). Because mosses lack an epidermal cuticle, the inhibition of nitrate reductase may contribute to the leaching of a fraction of the deposited nitrate instead of being stored in the tissues of these organisms. In contrast, the leaching of unassimilated nitrogen during excessive wet deposition is greatly prevented by the cuticle for vascular plants (Pitcairn *et al.*, 2006; Liu *et al.* 2012a,b).

Monitoring nitrogen deposition by means of different organisms can be a useful tool for estimating the rate of nitrogen deposition in many regions. However, caution must be taken because the inhibition of the nitrate reductase above a species-specific threshold can lead to underestimations of actual deposition rate.

## Perspectives

The nitrogen content and isotopic values of biomonitors can be suitable to inform environmental policy design for reducing the emissions of nitrogenous compounds, thus contributing to the mitigation of the adverse effects that atmospheric nitrogen deposition may have on priority ecosystems. Mosses can be especially useful because their nitrogen content responds directly to the rate of atmospheric deposition and their isotopic signature to the source. This is true up to certain deposition rate above which N accumulation decreases as a result of nitrate reductase inhibition. With the simultaneous use of different types of biomonitors, a multidimensional evaluation can be carried out regarding the state of ecosystems in the tropics. This could involve biomonitors that indicate the state of saturation, such as trees and shrubs, and those that indicate the source, such as vascular epiphytes with which it is possible to estimate the rate of atmospheric deposition using mosses. Further research should consider the ‘calibration’ and development of potential biomonitors suitable for each region of interest. For the case of tropical regions, atmospheric plants may prove particularly adequate. In any case, caution must be exercised given that biomonitors cannot provide the exact magnitude of atmospheric deposition, but a semiquantiative approximation, including characterizing the nitrogen source. In this case, the simultaneous use of an ensemble of various species can be of great utility in identifying areas subject to pollution by atmospheric nitrogen, especially in regions where nitrogen saturation has not occurred.

## References

[coy011C1] Ammann M, Siegwolf R, Pichlmayer F, Suter M, Saurer M, Brunold C (1999) Estimating the uptake of traffic-derived NO_2_ from ^15^N abundance in Norway spruce needles. Oecologia 118: 124–131. 28307686 10.1007/s004420050710

[coy011C2] Anderson KA, Downing JA (2006) Dry and wet atmospheric deposition of nitrogen, phosphorus and silicon in an agricultural region. Water, Air, Soil Pollut 176: 351–374.

[coy011C3] Andrade JL, de La Barrera E, Reyes-García C, Ricalde MF, Vargas-Soto G, Cervera JC (2007) El Metabolismo ácido de las crasuláceas diversidad, fisiología ambiental y productividad. Bol Soc Bot México 81: 37–51.

[coy011C4] Aneja V (2001) Atmospheric nitrogen compounds II: emissions, transport, transformation, deposition and assessment. Atmos Environ 35: 1903–1911.

[coy011C5] Bobbink R, Hicks K, Galloway J, Spranger T, Alkemade R, Ashmore M, Bustamante M, Cinderby S, Davidson E, Dentener F, et al. (2010) Global assessment of nitrogen deposition effects on terrestrial plant diversity: a synthesis. Ecol Appl 20: 30–59. 20349829 10.1890/08-1140.1

[coy011C6] Cameron KC, Di HJ, Moir JL (2013) Nitrogen losses from the soil/plant system: a review. Ann Appl Biol 162: 145–173. 10.1111/aab.12014.

[coy011C7] Cárdenas SMA, Delgadillo MC (2009) Musgos del Valle de México: Cuadernos 40. Instituto de Biología, Universidad Nacional Autónoma de México, México, D.F., pp 9–12.

[coy011C8] Conti ME, Cecchetti G (2001) Biological monitoring: lichens as bioindicators of air pollution assessment—a review. Environ Pollut 114: 471–492. 11584645 10.1016/s0269-7491(00)00224-4

[coy011C9] Craine JM, Brookshire ENJ, Cramer MD, Hasselquist NJ, Koba K, Marin-Spiota E, Wang L (2015) Ecological interpretations of nitrogen isotope ratios of terrestrial plants and soils. Plant Soil 396: 1–26.

[coy011C10] Craine JM, Elmore AJ, Aidar MPM, Bustamante M, Dawson TE, Hobbie EA, Kahmen A, Mack MC, McLauchlan KK, Michelsen A, et al. (2009) Global patterns of foliar nitrogen isotopes and their relationships with climate, mycorrhizal fungi, foliar nutrient concentrations, and nitrogen availability. New Phytol 183: 980–992. 19563444 10.1111/j.1469-8137.2009.02917.x

[coy011C11] Decina SM, Templer PH, Hutyra LR, Gately CK, Rao P (2017) Variability, drivers, and effects of atmospheric nitrogen inputs across an urban area: emerging patterns among human activities, the atmosphere, and soils. Sci Total Environ 609: 1524–1534. 28800694 10.1016/j.scitotenv.2017.07.166

[coy011C12] Díaz-Álvarez EA, de la Barrera E (2017) Mapping pollution in a megalopolis: the case for atmospheric biomonitors of nitrogen deposition. BioRxiv, 118257. 10.1101/118257.

[coy011C13] Díaz-Álvarez EA, Lindig-Cisneros R, de la Barrera E (2015) Responses to simulated nitrogen deposition by the neotropical epiphytic orchid Laelia speciosa. PeerJ 3: e1021. 26131375 10.7717/peerj.1021 PMC4485242

[coy011C14] Díaz-Álvarez EA, Reyes-García C, de la Barrera E (2016) A δ^15^N assessment of nitrogen deposition for the endangered epiphytic orchid Laelia speciosa from a city and an oak forest in Mexico. J Plant Res 129: 863–872. 27282994 10.1007/s10265-016-0843-y

[coy011C15] Ernshaw MJ, Winter K, Ziegler H, Stichler W, Cruttwell NE, Kerenga K, Cribb PJ, Wood J, Croft JR, Carver KA, et al. (1987) Altitudinal changes in the incidence of crassulacean acid metabolism in vascular epiphytes and related life forms in Papua New Guinea. Oecologia 73: 566–572. 28311975 10.1007/BF00379417

[coy011C16] Emmett BA (2007) Nitrogen saturation of terrestrial ecosystems: some recent findings and their implications for our conceptual framework. Water Air Soil Pollut Focus 7: 99–109.

[coy011C17] Emmett BA, Kjønaas OJ, Gundersen P, Koopmans C, Tietema A, Sleep D (1998) Natural abundance of ^15^N in forests across a nitrogen deposition gradient. For Ecol Manage 101: 9–18.

[coy011C18] Evans R (2001) Physiological mechanisms influencing plant nitrogen isotope composition. Trends Plant Sci 6: 121–126. 11239611 10.1016/s1360-1385(01)01889-1

[coy011C19] Felix JD, Avery GB, Mead RN, Kieber RJ, Willey JD (2016) Nitrogen content and isotopic composition of Spanish Moss (Tillandsia usneoides L.): reactive nitrogen variations and source implications across an urban coastal air shed. Environ Process 3: 711–722.

[coy011C20] Felix JD, Elliott EM (2014) Isotopic composition of passively collected nitrogen dioxide emissions: vehicle, soil and livestock source signatures. Atmos Environ 92: 359–366.

[coy011C21] Felix JD, Elliot EM, Gay DA (2017) Spatial and temporal patterns of nitrogen isotopic composition of ammonia at U.S. ammonia monitoring network sites. Atmos Environ 150: 434–442.

[coy011C22] Felix JD, Elliott EM, Gish T, Maghirang R, Cambal L, Clougherty J (2014) Examining the transport of ammonia emissions across landscapes using nitrogen isotopes ratios. Atmos Environ 95: 563–570.

[coy011C23] Felix JD, Elliott ME, Shaw SL (2012) Nitrogen isotopic composition of coal-fired power plant NOx: influence of emission controls and implications for global emission inventories. Environ Sci Technol 46: 3528–3535. 22288439 10.1021/es203355v

[coy011C24] Felix JD, Elliott EM, Timothy TJ, McConnell LL, Shaw SL (2013) Characterizing the isotopic composition of atmospheric ammonia emission sources using passive samplers and a combined oxidation-bacterial denitrifier approach. Rapid Commun Mass Spectrom 27: 2239–2246. 24019189 10.1002/rcm.6679

[coy011C25] Fenn ME, Baron JS, Allen EB, Rueth HM, Nydick KR, Geiser L, Bowman WD, Sickman JO, Meixner T, Johnson DW, et al. (2003) Ecological effects of nitrogen deposition in the Western United States. BioScience 53: 404–420.

[coy011C26] Forsum Å, Dahlman L, Näsholm T, Nordin A (2006) Nitrogen utilization by Hylocomium splendens in a boreal forest fertilization experiment. Funct Ecol 20: 421–426.

[coy011C27] Fowler D, Pyle JA, Raven JA, Sutton MA (2013) The global nitrogen cycle in the twenty-first century: introduction. Philos Trans R Soc B 368: 20130165. 10.1098/rstb.2013.0165 PMC3682749 23713127

[coy011C28] Freyer HD (1978) Seasonal trends of NH: and NO, nitrogen isotope composition in rain collected at Jiilich, Germany. Tellus 30: 83–92.

[coy011C29] Freyer HD (1991) Seasonal variation of ^15^N/^14^N ratios in atmospheric nitrate species. Tellus B 43: 30–44.

[coy011C30] Galloway JN, Detener FJ, Capone DG, Boyer EW, Howarth RW, Seitzinger SP, Asner GP, Cleveland CC, Green PA, Holland EA, et al. (2004) Nitrogen cycles: past, present, and future. Biogeochemistry 70: 153–226.

[coy011C31] Galloway JN, John D, Aber JD, Erisman JW, Seitzinger SP, Howarth RW, Cowling EB, Cosby BJ (2003) The nitrogen cascade. Bioscience 53: 341–356.

[coy011C32] Galloway JN, Townsend AR, Erisman JW, Bekunda M, Cai Z, Freney JR, Martinelli LA, Seitzinger SP, Sutton MA (2008) Transformation of the nitrogen cycle: recent trends, questions, and potential solutions. Science 320: 889–892. 18487183 10.1126/science.1136674

[coy011C33] Garten CT Jr (2006) Nitrogen isotope composition of ammonium and nitrate in bulk precipitation and forest throughfall. Int J Environ Anal Chem 47: 33–45.

[coy011C34] Gordon C, Wynn JM, Woodin SJ (2002) Impacts of increased nitrogen supply on high Arctic heath: the importance of bryophytes and phosphorus availability. New Phytol 149: 461–471. 10.1046/j.1469-8137.2001.00053.x 33873333

[coy011C35] Harmens H, Schnyder E, Thöni L, Cooper DM, Mills G, Leblond S, Mohr K, Poikolainen J, Santamaria J, Skudnik M, et al. (2014) Relationship between site-specific nitrogen concentrations in mosses and measured wet bulk atmospheric nitrogen deposition across Europe. Environ Pollut 194: 50–59. 25094057 10.1016/j.envpol.2014.07.016

[coy011C101] Harmens H, Norris DA, Cooper DM, Mills G, Steinnes E, Kubin E, Thöni L, Aboal JR, Alber R, Carballeira A, et al. (2011) Nitrogen concentrations in mosses indicate the spatial distribution of atmospheric nitrogen deposition in Europe. Environ Pollut 159: 2852–2860. 21620544 10.1016/j.envpol.2011.04.041

[coy011C36] Heaton THE (1990) ^15^N/^14^N ratios of NOx from vehicle engines and coal-fired power stations. Tellus B 42: 304–307.

[coy011C38] Heaton THE, Wynn P, Tye AM (2004) Low ^15^N/^14^N ratios for nitrate in snow in the High Arctic (79°N). Atmos Environ 38: 5611–5621.

[coy011C39] Heldt HW, Piechulla B (2011) Plant Biochemistry, Ed 4 Academic Press, London, England, pp 273–305.

[coy011C40] Hicks WK, Leith ID, Woodin SJ, Fowler D (2000) Can the foliar nitrogen concentration of upland vegetation be used for predicting atmospheric nitrogen deposition? Evidence from field surveys. Environ Pollut 107: 367–376. 15092983 10.1016/s0269-7491(99)00166-9

[coy011C41] Hietz P, Wanek W, Wania R, Nadkarni NM (2002) Nitrogen-15 natural abundance in a montane cloud forest canopy as an indicator of nitrogen cycling and epiphyte nutrition. Oecologia 131: 350–355. 28547706 10.1007/s00442-002-0896-6

[coy011C42] Hoering T (1957) The isotopic composition of the ammonia and the nitrate ion in rain. Geochim Cosmochim Acta 12: 97–102.

[coy011C43] Jones L, Provins A, Holland M, Mills G, Hayes F, Emmett B, Hall J, Sheppard L, Smith R, Sutton M, et al. (2014) A review and application of the evidence for nitrogen impacts on ecosystem services. Ecosyst Serv 7: 76–88.

[coy011C44] Kendall C, Elliott EM, Wankel SD (2007) Tracing anthropogenic inputs of nitrogen to ecosystems In Michener R, Lajtha K, eds Stable Isotopes in Ecology and Environment Science, Ed 2 Wiley-Blackwell, Oxford, pp 375–449.

[coy011C45] Kenkel JA, Sisk TD, Hultine KR, Sesnie SE, Bowker MA, Johnson NC (2016) Indicators of vehicular emission inputs into semi-arid roadside ecosystems. J Arid Environ 134: 150–159.

[coy011C46] Li D, Wang X (2008) Nitrogen isotopic signature of soil-released nitric oxide (NO) after fertilizer application. Atmos Environ 42: 4747–4754.

[coy011C47] Liu XY, Koba K, Liu CQ, Li XD, Yoh M (2012a) Pitfalls and new mechanisms in moss isotope biomonitoring of atmospheric nitrogen deposition. Environ Sci Technol 46: 12557–12566. 23050838 10.1021/es300779h

[coy011C48] Liu XY, Koba K, Makabe A, Li XS, Yoh M, Liu CQ (2013a) Ammonium first: natural mosses prefer atmospheric ammonium but vary utilization of dissolved organic nitrogen depending on habitat and nitrogen deposition. New Phytol 199: 407–419. 23692546 10.1111/nph.12284

[coy011C49] Liu XY, Koba K, Takebayashi Y, Liu CQ, Fang YT, Yoh M (2012b) Preliminary insights into δ^15^N and δ^18^O of nitrate in natural mosses: a new application of the denitrifier method. Environ Pollut 162: 48–55. 22243846 10.1016/j.envpol.2011.09.029

[coy011C50] Liu XY, Xiao HY, Liu CQ, Li YY, Xiao HW (2008a) Atmospheric transport of urban-derived NH(x): evidence from nitrogen concentration and δ^15^N in epilithic mosses at Guiyang, SW China. Environ Pollut 156: 715–722. 18644666 10.1016/j.envpol.2008.06.011

[coy011C51] Liu XY, Xiao HY, Liu CQ, Li YY, Xiao HW (2008b) Stable carbon and nitrogen isotopes of the moss *Haplocladium microphyllum* in an urban and a background area (SW China): the role of environmental conditions and atmospheric nitrogen deposition. Atmos Environ 42: 5413–5423.

[coy011C52] Liu XY, Xiao HY, Liu CQ, Li YY, Xiao HW (2008c) Tissue N content and ^15^N natural abundance in epilithic mosses for indicating atmospheric N deposition in the Guiyang area, SW China. Appl Geochem 23: 2708–2715.

[coy011C53] Liu X, Zhang Y, Han W, Tang A, Shen J, Cui Z, Vitousek P, Erisman J, Goulding K, Christie P, et al. (2013b) Enhanced nitrogen deposition over China. Nature 494: 459–462. 23426264 10.1038/nature11917

[coy011C54] Lu C, Tian H, Liu M, Ren W, Xu X, Chen G, Zhang C (2012) Effect of nitrogen deposition on China’s terrestrial carbon uptake in the context of multifactor environmental changes. Ecol Appl 22: 53–75. 22471075 10.1890/10-1685.1

[coy011C55] Markert BA, Breure AM, Zechmeister HG (2003) Definitions, strategies and principles for bioindication/biomonitoring of the environment In Markert BA, Breure AM, Zechmeister HG, eds Bioindicators & Biomonitors, Principles, Concepts and Applications. ELSEVIER, Oxford, pp 3–40.

[coy011C56] Moore H (1977) The isotopic composition of ammonia, nitrogen dioxide and nitrate in the atmosphere. Atmos Environ 11: 1239–1243.

[coy011C57] Morgan SM, Lee JA, Ashenden TW (1992) Effects of nitrogen oxides on nitrate assimilation in bryophytes. New Phytol 120: 89–97.

[coy011C58] Nakaji T, Fukami M, Dokiya Y, Izuta T (2001) Effects of high nitrogen load on growth, photosynthesis and nutrient status of *Cryptomeria japonica* and *Pinus densiflora* seedlings. Trees 15: 453–461.

[coy011C59] Ochoa-Hueso R, Allen EB, Branquinho C, Cruz C, Dias T, Fenn ME, Manrique E, Pérez-Corona ME, Sheppard MJ, Stock WD (2011) Nitrogen deposition effects on Mediterranean-type ecosystems: an ecological assessment. Environ Pollut 159: 2265–2279. 21277663 10.1016/j.envpol.2010.12.019

[coy011C60] Ollinger SV, Smith ML, Martin ME, Hallett RA, Goodale CL, Aber JD (2002) Regional variation in foliar chemistry and n cycling among forests of diverse history and composition. Ecology 83: 339–355.

[coy011C61] Padgett PE, Cook H, Bytnerowicz A, Heath RL (2009) Foliar loading and metabolic assimilation of dry deposited nitric acid air pollutants by trees. J Environ Monit 11: 75–84. 19137142 10.1039/b804338h

[coy011C62] Pardo LH, Templer PH, Goodale CL, Duke S, Groffman PM, Adams MB, Boeckx P, Boggs J, Campbell J, Colman B, et al. (2006) Regional assessment of N saturation using foliar and root. Biogeochemistry 80: 143–171.

[coy011C63] Payne RJ, Dise NB, Field CD, Dore AJ, Carpon SJ, Stevens CJ (2017) Nitrogen deposition and plant biodiversity: past, present, and future. Front Ecol Environ 15: 431–436.

[coy011C64] Pearson J, Wells DM, Seller KJ, Bennett A, Soares A, Woodall J, Ingrouille MJ (2000) Traffic exposure increases natural ^15^N and heavy metal concentrations in mosses. New Phytol 147: 317–326.

[coy011C65] Phoenix GK, Hicks WK, Cinderby S, Kuylenstierna CI, Stock WD, Dentener FJ, Giller KE, Austin AT, Lefroy DB, Gimeno BS, et al. (2006) Atmospheric nitrogen deposition in world biodiversity hotspots: the need for a greater global perspective in assessing N deposition impacts. Glob Change Biol 12: 470–476.

[coy011C66] Pinho P, Barros C, Augusto S, Pereira MJ, Máguas C, Branquinho C (2017) Using nitrogen concentration and isotopic composition in lichens to spatially assess the relative contribution of atmospheric nitrogen sources in complex landscapes. Environ Pollut 230: 632–638. 28711823 10.1016/j.envpol.2017.06.102

[coy011C67] Pitcairn C, Fowler D, Leith I, Sheppard L, Tang S, Sutton M, Famulari D (2006) Diagnostic indicators of elevated nitrogen deposition. Environ Pollut 144: 941–950. 16584821 10.1016/j.envpol.2006.01.049

[coy011C68] Pitcairn CER, Leith ID, Sheppard LJ, Sutton MA, Fowler D, Munro RC, Tang S, Wilson D (1998) The relationship between nitrogen deposition, species composition and foliar nitrogen concentrations in woodland flora in the vicinity of livestock farms. Environ Pollut 102: 41–48.

[coy011C69] Power SA, Collins CM (2010) Use of Calluna vulgaris to detect signals of nitrogen deposition across an urban–rural gradient. Atmos Environ 44: 1772–1780.

[coy011C70] Reyes-García C, Griffiths H (2009) Ecophysiological studies of perennials of the Bromeliaceae family in a dry forest: strategies for survival In de la Barrera E, Smith WK, eds Perspectives in Biophysical Plant Ecophysiology, A Tribute to Park S. Nobel. Universidad Nacional Autónoma de México, Ciudad de México, pp 121–151.

[coy011C71] Rosman KJR, Taylor PDP (1997) Isotopic compositions of the elements. Pure Appl Chem 70: 1593–1607.

[coy011C72] Sala OE, Chapin FS III, Armesto JJ, Berlow E, Bloomfield J, Dirzo R, Huber-Sanwald E, Huenneke LF, Jackson RB, Kinzing A, et al. (2000) Global biodiversity scenarios for the year 2100. Science 287: 1770–1774. 10710299 10.1126/science.287.5459.1770

[coy011C73] Schmidtlein S, Feilhauer H, Bruelheide H (2012) Mapping plant strategy types using remote sensing. J Veg Sci 23: 395–405.

[coy011C74] Sheng W, Yu G, Fang H, Liu Y, Wang Q, Chen Z, Zhang L (2014) Regional patterns of (15)N natural abundance in forest ecosystems along a large transect in eastern China. Sci Rep 4: 4249. 24576905 10.1038/srep04249 PMC3937787

[coy011C75] Shi XM, Song L, Liu WY, Lu HZ, Qi JH, Li S, Chen X, Wu JF, Liu S, Wu CS (2017) Epiphytic bryophytes as bio-indicators of atmospheric nitrogen deposition in a subtropical montane cloud forest: response patterns, mechanism, and critical load. Environ Pollut 229: 932–941. 28784334 10.1016/j.envpol.2017.07.077

[coy011C76] Simkin SM, Allen EB, Bowman WD, Clark CM, Belnap J, Brooksf ML, Cade BS, Collins SL, Geiser LH, Gilliam FS, et al. (2016) Conditional vulnerability of plant diversity to atmospheric nitrogen deposition across the United States. Proc Natl Acad Sci USA 15: 4086–4091. 10.1073/pnas.1515241113 PMC4839424 27035943

[coy011C77] Soderlund RSB (1976) The global nitrogen cycle. Ecol Bull 22: 23–73.

[coy011C78] Solga A, Burkhardt J, Zechmeister HG, Frahm JP (2005) Nitrogen content, ^15^N natural abundance and biomass of the two pleurocarpous mosses *Pleurozium schreberi* (Brid.) Mitt. and *Scleropodium purum* (Hedw.) Limpr. in relation to atmospheric nitrogen deposition. Environ Pollut 134: 465–473. 15620592 10.1016/j.envpol.2004.09.008

[coy011C79] Stewart G, Aidar MP, Joly CA, Schmidt S (2002) Impact of point source pollution on nitrogen isotope signatures (δ15N) of vegetation in SE Brazil. Oecologia 131: 468–472. 28547720 10.1007/s00442-002-0906-8

[coy011C80] Sulzman EW (2007) Stable isotope chemistry and measurement: a primer In Michener R, Lajtha K, eds Stable Isotopes in Ecology and Environmental Science, Ed 2 Blackwell Publishing Ltd, Oxford, pp 1–21.

[coy011C81] Sutton MA, Pitcairn CER, Whitfield CP (2004) Bioindicator and Biomonitoring Methods for Assessing the Effects of Atmospheric Nitrogen on Statutory Nature Conservation Sites, JNCC Report No: 356. Countryside Council for Wales, English Nature, Joint Nature Conservation Committee and Centre for Ecology and Hydrology. http://jncc.defra.gov.uk/pdf/jncc356.pdf (last accessed, 5 November 2016).

[coy011C82] Taiz L, Zeiger E (2002) Plant Physiology., Ed 3 Sinauer Associates, Sunderland, p 690.

[coy011C83] Vallano DM, Sparks JP (2008) Quantifying foliar uptake of gaseous nitrogen dioxide using enriched foliar δ^15^N values. New Phytol 17: 946–955. 10.1111/j.1469-8137.2007.02311.x 18069953

[coy011C84] Wanek W, Pörtl K (2008) Short-term 15N uptake kinetics and nitrogen nutrition of bryophytes in a lowland rainforest, Costa Rica. Funct Plant Biol 35: 51–62. 10.1071/FP07191 32688756

[coy011C85] Wang W, Pataki DE (2009) Spatial patterns of plant isotope tracers in the Los Angeles urban region. Landsc Ecol 25: 35–52.

[coy011C86] Wang W, Pataki DE (2011) Drivers of spatial variability in urban plant and soil isotopic composition in the Los Angeles basin. Plant Soil 350: 323–338.

[coy011C87] Wania R, Hietz P, Wanek W (2002) Natural ^15^N abundance of epiphytes depends on the position within the forest canopy: source signals and isotope fractionation. Plant, Cell Environ 25: 581–589.

[coy011C88] White JF Jr, Johnson H, Torres MS, Irizarry I (2012) Nutritional endosymbiotic systems in plants: bacteria function like “Quasi-Organelles” to convert atmospheric nitrogen into plant nutrients. J Plant Pathol Microb 3: e104.

[coy011C89] Widory D (2007) Nitrogen isotopes: Tracers of origin and processes affecting PM10 in the atmosphere of Paris. Atmos Environ 41: 2382–2390. 10.1016/j.atmosenv.2006.11.009.

[coy011C90] Wiedermann MM, Gunnarsson U, Ericson L, Nordin A (2009) Ecophysiological adjustment of two Sphagnum species in response to anthropogenic nitrogen deposition. New Phytol 181: 208–217. 18811618 10.1111/j.1469-8137.2008.02628.x

[coy011C91] Xiao HY, Liu CQ (2002) Sources of nitrogen and sulfur in wet deposition at Guiyang, Southwest China. Atmos Environ 36: 5121–5130.

[coy011C92] Xiao HY, Tang CG, Xiao HW, Liu XY, Liu CQ (2010) Mosses indicating atmospheric nitrogen deposition and sources in the Yangtze River Drainage Basin, China. J Geophys Res 115: D14301.

[coy011C93] Xiao HW, Xiao HY, Long AM, Wang YL (2012) Who controls the monthly variations of NH4+ nitrogen isotope composition in precipitation? Atmos Environ 54: 201–206.

[coy011C94] Zambrano A, Medina C, Rojas A, López D, Chang L, Sosa G (2009) Distribution and sources of bioaccumulative air pollutants at Mezquital Valley, Mexico, as reflected by the atmospheric plant Tillandsia recurvata L. Atmos Chem Phys 9: 6479–6494.

[coy011C102] Zechmeister HG, Richter A, Smidt S, Hohenwallner D, Roder I, Maringer S, Wanek W (2008) Total nitrogen content and δ15N signatures in moss tissue: indicative value for nitrogen deposition patterns and source allocation on a nationwide scale. Environ Sci Technol 42: 8667–8667. 10.1021/es801865d 19192778

[coy011C95] Zhang R, Zhou Z, Luo W, Wang Y, Feng Z (2013) Effects of nitrogen deposition on growth and phosphate efficiency of *Schima superba* of different provenances grown in phosphorus-barren soil. Plant Soil 370: 435–445.

[coy011C96] Zotz G, Wiebke B, Hietz P, Nadine K (2010) Growth of epiphytic bromeliads in a changing world: The effects of CO_2_, water and nutrient supply. Acta Oecol 36: 659–665.

